# Breastfeeding and Maternal Perceptions of Infant Sleep, Settle and Cry Patterns in the First 9 Months

**DOI:** 10.3390/ijerph192013098

**Published:** 2022-10-12

**Authors:** Sharon Lisa Perrella, Alice Dix-Matthews, Julie Williams, Alethea Rea, Donna Tracy Geddes

**Affiliations:** 1 School of Molecular Sciences, The University of Western Australia, Crawley, WA 6009, Australia; 2 School of Medicine, The University of Western Australia, Crawley, WA 6009, Australia; 3 Neonatology Clinical Care Unit, King Edward Memorial Hospital, Subiaco, WA 6008, Australia; 4 Mathematics and Statistics, Murdoch University, Murdoch, WA 6150, Australia

**Keywords:** sleep behaviours, child development, infant sleep, maternal bother, breastfeeding

## Abstract

This study evaluated relationships between maternal perceptions of infant sleep, settling and crying patterns and breastfeeding. A prospective observational study of 91 mothers of healthy, term infants was conducted with follow ups over 9 months after discharge from a Western Australian maternity hospital. Feeding information, sleep, settle and cry behaviours, maternal bother at infant behaviours and confidence were measured using the Sleep and Settle Questionnaire. Breastfeeding confidence was measured using the Breastfeeding Self-Efficacy Scale—Short Form. Questionnaires were administered at 2 and 6 weeks, 3, 6 and 9 months. Linear mixed models were used to assess associations between maternal bother, feeding method and infant characteristics. The feeding method was not associated with maternal bother, and cessation of breastfeeding did not result in a change in bother scores (*p* = 0.34). Duration of infant crying in the day, evening and night, frequency of night waking and duration of settling to sleep in the day were associated with increased bother scores. Higher breastfeeding self-efficacy and maternal confidence were associated with lower bother scores (both *p* < 0.01). Maternal bother is associated with infant behaviours that require parental input, but not breastfeeding status. Resources that address parental expectations regarding infant sleep while providing strategies to support maternal wellbeing and breastfeeding are needed.

## 1. Introduction

Parents’ concerns about their child’s sleep are highest in the first year of life [1], with approximately a third of parents rating their infants’ sleep as problematic [2,3,4]. An understanding of parents’ perceived infant sleep problems is critical as they can negatively impact parents, being associated with decreased emotional stability, fatigue and marital difficulty [5,6,7]. The most common reported concerns are frequent night waking, difficulty settling to sleep and total night sleep duration [2,3,8,9]. There is, however, a wide variation in normal infant sleep, settling and cry patterns in the first 12 months [4], and not all parents are bothered by the same reported infant behaviours. Perceptions of problematic infant sleep are shaped by parents’ own confidence with managing infant sleep [10], antenatal and postnatal maternal mental health [11,12] and maternal sleep quality and daytime functioning [2,8,13].

Breastfeeding and infant sleep are entwined, with shorter sleep–wake cycles supporting frequent feeding in the early months, and hormones such as melatonin in breast milk aiding the infant’s return to sleep [14]. However, the interplay between breastfeeding and infant sleep behaviours, such as night waking frequency and sleep duration, is unclear. A recent systematic review has highlighted inconsistent evidence of differences in night waking frequency and night sleep duration between breastfed and formula-fed infants [15]. Despite this, some mothers are encouraged to cease breastfeeding as a management strategy to improve infant sleep [16].

Similarly, evidence for a link between breastfeeding and perceived infant sleep problems is inconsistent [3,9,17,18,19]. To better understand the relationship between breastfeeding and maternal perceptions of infant sleep, we evaluated associations between the feeding method and maternal perceptions of infant sleep, settle and cry patterns, and their degree of bother relating to these over the first 9 months postpartum.

## 2. Materials and Methods

A prospective observational longitudinal cohort study of feeding practices and sleeping patterns was conducted across the first 9 months postpartum in cohorts of mothers with healthy term infants (birth ≥ 37 weeks gestation, with no neonatal unit admissions) and preterm/sick infants. The aims of the study were to describe and compare the following outcomes of the two cohorts at hospital discharge and across the first 9 months post-discharge: breastfeeding rates, maternal perceptions of infant sleeping and settling behaviours and maternal breastfeeding confidence and satisfaction. This paper reports the outcomes of the healthy term cohort; outcomes of the preterm/sick cohort will be reported and compared in another paper.

An estimated sample size of 70 mothers was required to detect a 25% difference in breastfeeding rates with 86% power. To account for attrition, we recruited 96 mothers of healthy term infants. Participants were recruited from King Edward Memorial Hospital’s postnatal wards in Perth, Western Australia, between October 2006 and July 2007, and followed up via telephone interview at 2 and 6 weeks, 3, 6 and 9 months after discharge. Inclusion criteria were English-speaking mothers, 18 years or older, who were breastfeeding and/or expressing breast milk for their infant at the time of discharge from hospital and intended to continue. The Ethics Committee of the Women’s & Children’s Health Service approved the study (reference EC06-05). Prospective participants were provided with verbal and written study information and informed written consent was obtained.

At recruitment, demographic data were collected, and a breastfeeding questionnaire was administered, with items including previous breastfeeding duration and intended breastfeeding duration. Socio-economic status was determined by Socio-economic Index for Areas (SEIFA) that ranks relative socio-economic disadvantage by postal code [20].

At each follow up time point mothers reported their current infant feeding method (fully breastfeeding: no food or fluids other than breast milk, mixed feeding: breast milk and formula and/or other fluids or food, or fully formula feeding), timing of introduction of solids and date of breastfeeding cessation.

The Breastfeeding Self-Efficacy Scale-Short Form (BSES-SF) [21] was used to measure mothers’ confidence in their ability to breastfeed by rating 14 items using a 5-point Likert scale (1 = not confident; 5 = very confident). Ratings are summed to give a total score (range 14 to 70), where higher scores indicate higher breastfeeding confidence. The BSES-SF has high reliability with a Cronbach’s α coefficient of 0.94 [21]. It was completed at recruitment and at each follow up time point, or until cessation of breastfeeding.

The Sleep and Settle Questionnaire [22] recorded maternal perceptions of infant sleep and settling behaviours, and maternal level of ‘bother’ or concern with these behaviours at each follow up time point. Parents report their infant’s typical behaviours over the last week, including durations of crying, settling and sleep, and frequencies of day sleeps and night waking. Infant behaviours are reported for morning (0500 h to 1200 h) and afternoon (1200 h to 1800 h) or daytime (0500 h to 1800 h), as well as evening (1800 h to 2200 h) and night-time (2200 h to 0500 h). Nine of the thirty-four items are rated to indicate the degree of bother with infant sleep, settle and cry behaviours using a 5-point Likert scale (1 = did not bother me at all; 5 = bothered me extremely). Ratings are summed to give a total bother score (range 9 to 45), where higher scores indicate a higher degree of bother. Two items assess maternal confidence in the ability to settle their baby, summed to give a total confidence score (range 2 to 10), and one item assesses maternal confidence in their partner’s ability to calm or settle their baby (range 1 to 5). The SSQ has adequate test–retest reliability (“moderate” 0.14–0.76) [22].

As we were primarily interested in maternal perceptions of infant behaviours, no attempt was made to address bias that is inherent in subjective reports of behaviour.

Demographic and background feeding characteristics and feeding methods are described using frequency and percentage for categorical variables and mean and standard deviation for continuous variables. Descriptive statistics for sleep, settle and cry behaviours, total bother score, and BSES-SF score are presented as mean and standard deviation for each time point. We used R (R Foundation Statistical Computing, 2022, Vienna, Austria) for all analyses, with the statistical significance level set at 0.05.

A linear mixed model was fitted with total bother as the response and a random effect for mother. We examined associations between time (a categorical variable for each follow-up time point) and 13 fixed effect explanatory variables: feeding method, sleep variables (morning, afternoon, evening and night sleep; day sleep frequency; night waking frequency), settling and crying durations for daytime, evening and night. Thirteen co-founders were considered: maternal age, maternal education, marital status, ancestry, birth gestation, parity, intended breastfeeding duration, BSES-SF score, total confidence score and confidence in partner score.

Survival modelling investigated the time to ceasing breastfeeding with five explanatory variables: total bother score; maternal age; maternal education; parity; and intended duration of breastfeeding. The change in total bother score over time was explored visually and for those that reported cessation of breastfeeding, a t-test was used to investigate pre-post cessation changes in bother scores.

## 3. Results

Of the 110 eligible mothers that were approached, 96 mothers of healthy term infants were recruited. We excluded 4 mothers of twin infants from the analysis due to the differing experiences and confidence reported in parents of twins [23], and one mother withdrew at 2 weeks due to fatigue, resulting in a sample of 91 mothers. Fourteen mothers (15%) were lost to follow-up at the 9-month time point. Maternal demographic and background feeding characteristics are reported in Table 1.

Rates of ‘any’ and ‘full’ breastfeeding reduced from 96% and 82% at 2 weeks to 70% and 57% at 6 months, respectively, with 59% still breastfeeding at 9 months (Table 2). Mean BSES-SF score was 54.6 at recruitment and increased to 64 at 9 months (Table 1 and Table 2).

Maternal reports of infant sleep, settle and cry behaviours across the first 9 months are summarized in Table 3. Duration of morning sleep showed little change across the 9 months while afternoon sleep duration decreased, and night sleep duration increased. Night waking was reported at all time points and decreased over time. Mothers reported spending more time settling their infant to sleep at 2 and 6 weeks, than at subsequent time points. Crying durations in the day, evening and night all showed large inter-individual variability, with a downward trend over time.

Across the first 9 months, mothers typically reported a low degree of bother, and had high levels of confidence in themselves and in their partners regarding the ability to soothe their unsettled infant (Table 3). Survival modelling found that total bother score was not associated with time to breastfeeding cessation, however intended breastfeeding duration was positively associated with actual breastfeeding duration (*p* = 0.002). Pre-post breastfeeding cessation bother scores were not significantly different (t(29) = −0.97, *p* = 0.34).

Higher breastfeeding confidence had a small but significant association with lower bother scores, while a higher maternal educational level was associated with higher bother scores (Table 4).

**Figure 1:**
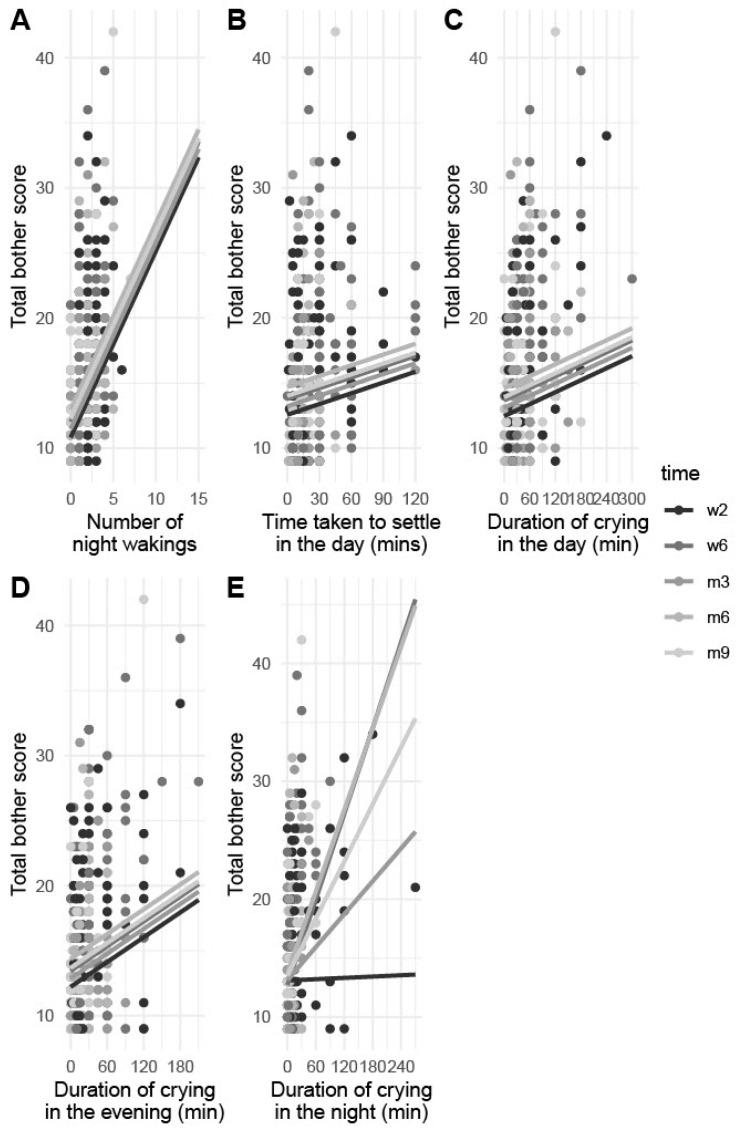
Linear mixed models showing the relationship between total bother scores and night waking frequency (**A**), time taken to settle in the day (**B**), duration of crying in the day (**C**), evening (**D**) and night (**E**).

Using linear mixed modelling we found that increased frequency of night waking (Figure 1A), increased time taken to settle in the day (Figure 1B) and duration of crying in the day (Figure 1C), and evening (Figure 1D) were associated with increased total bother scores (Table 4). The duration of crying at night was associated with an increase in total bother scores at all time points, except for at 2 weeks where it was equivocal (Figure 1E). All other variables considered were not associated with total bother.

## 4. Discussion

In our evaluation of the relationships between maternal bother and maternal perceptions of infant crying, settling and sleep, we found associations with night waking frequency, and durations of crying and daytime settling (Table 4). Maternal bother in relation to infant crying, settling and sleep behaviours was not associated with breastfeeding, contrasting with studies that have associated breastfeeding with increased likelihood of negative maternal perceptions of infant sleep [3,17,18]. Instead, infant night waking and crying were most consistently associated with increased maternal bother across the first 9 months postpartum. This finding is congruent with previous studies showing that night waking frequency, particularly, is a key intrinsic factor associated with parental perceptions of problematic infant sleep [2,3,8,9].

Our maternal reports of infant sleep, settling and crying patterns are consistent with values previously reported [24,25,26]. We found that maternal bother was not associated with feeding type, and did not reduce after ceasing breastfeeding, indicating that maternal concern for their infant’s sleep is independent of their choice of feeding type in the first 9 months postpartum. Knowledge gaps with regard to normal infant sleep physiology may have influenced the degree of bother experienced by these mothers. Our cohort had low bother scores, high confidence in themselves and their partners with regard to the ability to settle their baby, and high breastfeeding confidence throughout (Table 3). Mean total bother scores were <18 across all time points (score range 9 to 45), and mean BSES-SF scores were ≥60 after discharge from hospital (score range 14 to 70, low confidence < 58) [27]. The early breastfeeding success of mothers in this cohort is likely explained by high breastfeeding self-efficacy and longer breastfeeding intentions that are both associated with continued breastfeeding, while other constructs of the Theory of Planned Behaviour such as attitudes and subjective norms have little to no effect [27,28]. It is possible that associations between maternal bother, feeding method and infant settling and sleep may differ for women with low breastfeeding confidence and/or high degrees of bother. Future study should focus on higher risk populations, such as those with pre-existing mental health challenge [12].

Previous research has reported a positive association between breastfeeding and perceptions of problematic sleep, however, the studies have predominantly focused on late infancy (~12 months) [3,17], while our study focused on the first 9 months. A recent systematic review [15] concluded that, early in infancy (<6 months) breastfed infants had comparable night sleep duration and frequency of night waking to formula fed infants, while in later infancy (>6 months), breastfeeding was associated with a decreased total night sleep duration. It is possible those breastfeeding at ~12 months may be more likely to find their infants’ sleep problematic, compared to those breastfeeding in early infancy. Further longitudinal studies which include both early and late infancy are needed to determine the temporal nature of the association between breastfeeding and perceived infant sleep problems. Regardless, our findings encourage clinicians to look beyond feeding method when supporting parents reporting problematic infant sleep.

In this study, infant behaviours such as crying and night waking were associated with increased bother (Figure 1). Frequency of night waking, duration of day and evening crying and time taken to settle in the day were positively associated with total bother scores throughout, while duration of night crying was equivocal at 2 weeks then positively associated afterwards (Table 4). At 2 weeks postpartum, parents may expect increased infant crying, allowing for greater tolerance which may diminish over time. Still, waking frequently at night is incongruent with biologically normal adult sleep patterns, so it seems reasonable that parents would report infant night waking and crying to be most problematic, despite both being common and biologically normal for infants [4]. Maternal bother regarding settling duration during the day makes sense clinically, as an infant’s need for attention during the day can make it difficult to complete any tasks [22]. Our findings are consistent with previous studies showing infant crying and night waking to be the most common issues for which parents present to health professionals, and so contribute significantly to healthcare costs for infants under 12 weeks of age [29]. These behaviours are associated with increased maternal postnatal fatigue and depressive symptoms [30,31,32].

However, the relationship between infant sleep and maternal mental health is bi-directional [11]. Women with antenatal mental health challenges are at higher risk of reporting difficulties with infant sleep [11,33], reflected in increased rates of perceived infant sleep problems [11,12]. Perceptions and reporting of sleep problems in young children are negatively associated with parental confidence relating to children’s sleep [10]. Mothers with lower confidence in their parenting abilities may see a broader range of infant sleep behaviours as problematic, while mothers with higher confidence may be able to better identify normal infant sleep behaviours. It is important to consider parenting confidence and mental health status when examining perceptions of infant sleep, and greater support should be provided to these parents. Traditional infant sleep education programs are not consistently effective and are unacceptable to some parents [34]. An alternative approach is a responsive sleep intervention, with emerging evidence of effectiveness in consolidating infant sleep and reducing night waking while promoting maternal adjustment and reducing rates of perceived problematic infant sleep [34,35,36].

Limitations: In this study, infant sleep was assessed subjectively, using self-report questionnaires, rather than objective methods such as actigraphy. The advantage of self-report data is that it identifies infant behaviours that are most likely to illicit parental bother. For example, only infant night waking that also wakes the parents is reported. However, parental reports are known to over-estimate sleep duration, and underestimate night waking frequency [37,38]. Maternal mental health is known to impact maternal sleep and perceptions of infant sleep [11,12]. Our study did not record perinatal mental health status, and this important factor should be included in future studies. Only a small number of our participants ceased breastfeeding in the first 3 months, limiting the power of comparisons by feeding group in the early months after birth. Birth mode was not recorded for this study. A longitudinal comparison of infant sleep outcomes and human milk concentrations of melatonin by birth mode would elucidate whether a likely reduction in colostrum and early milk concentrations of melatonin after Caesarean birth has longer term impacts on infant sleep [39,40]. The timing of breast expressions and feeding of that milk was not recorded. Future studies should consider the potential impact of mis-timed feeding of milk, as human milk concentrations of melatonin and cortisol have circadian patterns that may shape infant sleep patterns [14]. Finally, we assessed a cohort of primarily partnered, Caucasian, educated mothers with high breastfeeding confidence, which limits the extrapolation of these findings to diverse populations. Future exploration of strategies used by these women to successfully establish and continue lactation will provide valuable information to inform breastfeeding education and care.

## 5. Conclusions

Infant sleep is a major concern to many new parents and can cause significant distress. We found that while breastfeeding is not associated with increased maternal bother regarding their infant’s sleep, increased nocturnal infant waking, crying and time taken to settle back to sleep had modest positive associations with maternal bother across the first 9 months. Our findings add to the limited body of evidence on breastfeeding and perceived infant sleep problems and suggest that how parents cope with infant night-time interruptions and crying, regardless of their feeding type, contributes to their perception of problematic infant sleep. Future studies should focus on clarifying this relationship in high-risk populations, especially mothers with limited support and with pre-existing mental health challenges.

## Figures and Tables

**Table 1:** Maternal demographic and background feeding characteristics.

Characteristics	*n* (%)	M ± SD
Age < 30 years	41 (45)	
SEIFA decile ^1^		6.9 ± 2.7
Primipara	50 (54)	
Married/de facto	83 (90)	
Caucasian	76 (84)	
Infant sex = female	44 (49)	
Education level		
High school	32 (35)	
TAFE ^2^/diploma	31 (34)	
High school	29 (32)	
Caesarean birth	39 (43)	
Birth gestation, weeks		39.7 ± 1.3
Hospital stay, days		3.8 ± 1.2
Intended breastfeeding duration		46 ± 24
BSES-SF score at discharge		54.6 ± 10.1

^1^ SEIFA, Socio-Economic Index For Areas, Index of Relative Socio-Economic Disadvantage; Australian census data are used to rank areas by postal code; higher deciles reflect higher levels of socio-economic advantage. (20) ^2^ TAFE, technical and further (non-tertiary) education.

**Table 2:** Feeding outcomes across the first nine months.

Feeding Outcomes	2 Weeks	6 Weeks	3 Months	6 Months	9 Months
BSES-SF score (M ± SD)	*n* = 83	*n* = 70	*n* = 65	*n* = 51	*n* = 41
	60 ± 9.9	62 ± 8.3	62 ± 8.8	65 ± 6.3	64 ± 6.9
Feeding method *n* (%)	*n* = 85	*n* = 83	*n* = 81	*n* = 75	*n* = 73
Fully breastfeeding	70 (82)	64 (77)	50 (62)	43 (57)	23 (32)
Mixed feeding	12 (14)	12 (14)	17 (21)	10 (13)	20 (27)
Formula feeding	3 (4)	7 (8)	14 (17)	22 (29)	30 (41)
Introduced solids	0 (0)	0 (0)	1 (1)	64 (89)	76 (100)

**Table 3:** Sleep and Settle Questionnaire (SSQ) outcomes across the first nine months.

SSQ Items (M ± SD)	2 Weeks	6 Weeks	3 Months	6 Months	9 Months
	(*n* = 88)	(*n* = 83)	(*n* = 80)	(*n* = 74)	(*n* = 77)
Duration of sleeps, h					
Morning sleeps	2.5 ± 0.9	2.2 ± 1.1	2.1 ± 1.1	2.1 ± 1.1	2.3 ± 1.1
Afternoon sleeps	2.4 ± 1.0	2.1 ± 1.1	1.9 ± 1.0	1.7 ± 0.8	1.2 ± 0.6
Evening sleeps	1.8 ± 1.1	1.8 ± 1.0	1.6 ± 1.0	2.1 ± 1.1	2.2 ± 0.9
Night sleeps	3.4 ± 1.3	4.1 ± 1.5	5.4 ± 1.6	5.9 ± 1.5	6.6 ± 0.9
Night-waking frequency	2.4 ± 1.1	1.8 ± 1.0	1.1 ± 1.1	1.2 ± 1.3	1.4 ± 2.1
Day-time sleep frequency	4.0 ± 1.2	3.4 ± 1.2	3.1 ± 0.8	2.7 ± 0.8	2.3 ± 1.6
Duration of settling to sleep, min					
Daytime	25 ± 24	30 ± 30	18 ± 17	14 ± 10	13 ± 10
Evening	36 ± 41	35 ± 34	20 ± 16	18 ± 16	15 ± 14
Night	30 ± 33	20 ± 23	10 ± 15	8.7 ± 10	8.8 ± 10
Crying duration, min					
Daytime	45 ± 48	55 ± 49	39 ± 34	40 ± 34	37 ± 36
Evening	31 ± 40	42 ± 42	25 ± 22	23 ± 21	18 ± 20
Night	28 ± 44	13 ± 16	5.5 ± 10	6.8 ± 8.0	9.8 ± 15
Total bother score	17 ± 5.9	17 ± 6.9	14 ± 4.6	14 ± 5.4	14 ± 5.4
Total confidence score	8.8 ± 1.1	8.9 ± 1.1	9.2 ± 1.0	9.6 ± 0.9	9.5 ± 0.9
Total confidence in partner score	3.6 ± 1.0	3.4 ± 1.0	3.5 ± 1.0	3.8 ± 1.0	4.0 ± 1.3

Morning (0500 h to 1200 h), Afternoon (1200 h to 1800 h), Evening (1800 h to 2200 h), Night (2200 h to 0500 h); Daytime (0500 h to 1800 h), Evening (1800 h to 2200 h), Night (2200 h to 0500 h).

**Table 4:** Linear mixed models using feed, sleep, settle and cry variables to predict total bother scores.

Fixed Effects	Estimate	SE	*p*-Value	Anova *p*-Value
(Intercept)	22.12	3.50	<0.01	(Intercept)
Time (6 weeks)	−0.38	0.78	0.63	0.05
Time (3 months)	0.050	0.78	0.95	
Time (6 months)	0.63	0.91	0.49	
Night-waking frequency	1.44	0.22	<0.01	
Duration of time taken to settle (day)	0.03	0.012	0.02	
Duration of crying (day)	0.028	0.007	0.02	
Duration of crying (evening)	0.032	0.009	<0.01	
Duration of crying (night)	0.002	0.011	0.86	
Maternal education	1.46	0.39	<0.01	
BSES-SF score	−0.10	0.034	<0.01	
Total confidence score	−1.11	0.28	<0.01	
Time (6 weeks): Duration of crying (night)	0.12	0.033	<0.01	<0.01
Time (3 months): Duration of crying (night)	0.045	0.049	0.36	
Time (6 months): Duration of crying (night)	0.11	0.063	0.07	
Time (9 months): Duration of crying (night)	0.079	0.038	0.04	

## Data Availability

The data that support the findings of this study are available from the corresponding author (SP) upon reasonable request.
